# Supplementary material for: Personalized bacteriophage therapy outcomes for 100 consecutive cases: a multicentre, multinational, retrospective observational study
Source: Nat Microbiol. 2024 Jun 4;9(6):1434–53. doi: 10.1038/s41564-024-01705-x (PMC11153159; doi:10.1038/s41564-024-01705-x)
Supplement: Supplementary file 1 — Supplementary Tables 1–4. [file 41564_2024_1705_MOESM1_ESM.pdf]

nature microbiology

Supplementary information

https://doi.org/10.1038/s41564-024-01705-x

Personalized bacteriophage therapy outcomes for 100 consecutive cases: a multicentre, multinational, retrospective observational study

In the format provided by the authors and unedited

Supplementary Table 1 | Overview of the first 100 consecutive bacteriophage therapy cases facilitated by a Belgian consortium, displayed in chronological order

	Infection						Bacteriophage product			Treatment										Reported adverse events						Clinical outcome			
Patient #	Primary infection type	Secondary/ concomitant infection type	Acute or chronic infection	Additional information	Involved bacterial species	Antibiotic resistance profile of the targeted strain(s)	Bacteriophage(s)	Diluent	Concentration (PFU/ml)	Intraoperative	Administration route 1	Dosage	Duration	Administration route 2	Dosage	Duration	Concomitant antibiotic treatment (route of administration)	Bacteriophage therapy prematurely stopped	Ambulatory treatment or hospitalised	AE	Duration	Severity	Relationship to bacteriophage treatment	Action	Patient outcome	Clinical improvement	Eradication of the targeted bacteria	Clinical comment	Regulatory context
1	BoneI		Chronic	Chronic pelvic osteomyelitis	Proteus mirabilis, Morganella morganii and Staphylococcus aureus*	MDR	BFC 1	PBS	10^7	20 ml (absorbed into Duracol)	IL (draining system)	20 ml q24h	5 days				Ciprofloxacin (IV), rifampicin (IV), and linezolid (IV)	No	Hospital	No						Yes	Yes	Osteomyelitis resolved	H
2	BoneI		Chronic	Osteomyelitis of the foot	S. aureus*	UDR	BFC 1	NaCl 0.9%	10^7		IL, injection (n=3) or basorbed in compress bandage (n=1), after curettage	In excess, 4 applications spread over a period of 1 year	1 year				Ciprofloxacin (oral)	No	Hospital	No						No	No	Amputation	H
3	LRTI		Acute	Pulmonary septic shock due to influenza	Pseudomonas aeruginosa*, Haemophilus influenzae	XDR	BFC 1	PBS	10^7		Nebulisation	Unknown	4 days				Meronem (IV), tobramycin (IV), and colymicine (aerosol)	Yes	Hospital	Pulmonary septic shock	NA	Severe	Unlikely	Bacteriophage therapy stopped	Died	No	No	Patient died due to a pulmonary septic shock	H
4	BoneI		Chronic	Chronic osteomyelitis with fistula sacrum	P. aeruginosa*	MDR	BFC 1	NaCl 0.9%	10^7		Topical (bandages soaked with bacteriophages)	30 ml q8h	3 weeks				Ciprofloxacin (oral) and rifampicin (oral)	No	Hospital	No						Yes	Yes		H
5	Other		Chronic	Prostatitis	Escherichia coli*	MDR	PyoPhage	Undiluted	Unknown		Rectal	10 ml q12h	1week				Fosfomycin (oral)	No	Ambulatory	No						Yes	Yes	No more clinical signs of prostatitis	H
6	LRTI		Chronic	CF lung infection	E. coli* and P. mirabilis*	XDR (E coli) and MDR (P mirabilis)	IntestiPhage	Undiluted	Unknown		Nebulisation	4 ml q6h	10 days					No	Hospital	No						Yes	Yes		H
7	URTI		Chronic	Chronic sinusitis	P. aeruginosa*	UDR	BFC 1	PBS	10^7		Nasal spray	25 ml q8h	3 weeks					No	Ambulatory	No						Yes	Yes		H
8	LRTI	UTI	Acute + chronic	Pulmonary and urinary sepsis in a heart transplanted patient	P. aeruginosa*	XDR	BFC 2	NaCl 0.9%	10^7		Nebulisation	2 ml q6h (day 1) followed by 4 ml q6h (days 2-14)	2 weeks	Rectal (short rectal canule)	10 ml q24h (> 1h after stool, followed by lying down for 30 min)	4 weeks	Colistin (IV) and ceftolozane/tazobactam (IV)	No	Hospital	No						Yes	Yes	Patient transferred for revalidation	H
9	BoneI		Chronic	Osteomyelitis of the left hip and femur	Klebsiella pneumoniae*	XDR	M1	Undiluted	10^8	100 ml (10 x diluted)	IL (draining system)	20 ml q8h	6 days				Ceftazidime/avibactam (IV), moxifloxacin (IV), and tigecyclin (IV)	No	Hospital	No						Yes	Yes	Osteomyelitis resolved	H
10	URTI		Chronic	Skin infection with large furuncles and nasal colonisation	S. aureus*	UDR	BFC 2	PBS	10^7		Nasal spray	1 ml q12h	1 week					No	Ambulatory	No						Yes	Yes	Decrease in furuncle quantity and frequency, bacterial pathogens were no longer isolated from nose, perineum, rectal and throath swabs, but 6 months after bacteriophage treatment furuncles re-emerged	H
11	LRTI		Chronic	Primary ciliary dyskinesia with bronchiectasis	P. aeruginosa*	MDR	BFC 2	NaCl 0.9%	10^7		Nebulisation	q12h (1 ml day 1, 2 ml day 2, 3 ml day 3, 4 ml day 4, 5 ml days 5-10)	10 days	Oral	20 ml (after 10 ml Maalox) q24h	10 days		No	Hospital	Coughing after product inhalation	6 days	Moderate	Possible	None	Recovered	Yes	No	Improved breathing	H
12	LRTI		Chronic	Primary ciliary dyskinesia with bronchiectasis	P. aeruginosa*	UDR	BFC 2	NaCl 0.9%	10^7		Nebulisation	2 ml q6h (day 1) followed by 4 ml q6h (days 2-11)	11 days					No	Hospital	No						Yes	Yes	Less oxygen needed, inflammation resolved	H
13	SSTI	BSI	Acute + chronic	Infection of sacral and spinal back pressure sores with sepsis	P. aeruginosa*	XDR	BFC 1	NaCl 0.9%	10^6		IV	50 ml (6 h infusion) q24h	10 days					No	Hospital	No						Yes	Yes	Patient deceasedseveral months after bacteriophage therapy due to a septic shock caused by another bacterial pathogen	H
14	SSTI		Chronic	Chronic venous ulcer	S. aureus* and P aeruginosa*	MDR	BFC 2	PBS	10^7		Topical	125 ml q24h	8 days					No	Hospital	No						No	No		H
15	OPI		Acute	Septic arthritis of the prosthetic knee	P. aeruginosa*	UDR	14-1, PNM, and PT07	NaCl 0.9%	10^7	50 ml during arthroscopy							Ceftazidime (IV) and ciprofoxacin (oral)	No	Hospital	No						Yes	Yes		H
16	LRTI		Acute	Pneumonia in double lung-transplanted CF patient	Achromobacter xylosoxidans*	PDR	APC 1.1 and APC 2.1	NaCl 0.9%	10^8-10^9		Nebulisation	5 ml q8h	APC 1.1 for 2 days and, 17 weeks later, APC 2.1 for 2 weeks	Lung instillation (fibroscope)	APC 2.1, one dose, 30 ml per lobe	1 day		No	Hospital	No						Yes	Yes	Increased pulmonary function	ATU MP
17	LRTI		Chronic	Cartilage hair hypoplasia syndrome with recurrent pulmonary and gastro-intestinal infections and bronchiectasis	P aeruginosa*	MDR	BFC 2	PBS	10^7		Nebulisation	45 ml q12h (days 1-10) followed by 5 ml q12h (days 11-28)	4 weeks	Oral	20 ml q12h	4 weeks	Colistin (aerosol) and meropenem (IV)	No	Hospital	No						Yes	Yes	Patient deceased later due to a septic shock caused by another bacterial pathogen	H
18	SSTI		Chronic	Chronic wound infection	P aeruginosa*	XDR	BFC 2	PBS	10^7		Topical	100 ml q24h	10 days					No	Hospital	No						Yes	Yes	Wounds completely closed, patient regained ambulation and was discharged	SOC MP
19	SSTI		Acute	Burn wounds on back and bottom (TBSA burbed: 30%, 2nd and 3rd degree)	Acinetobacter baumannii*, K pneumoniae, Staphylococcus haemolyticus, and P mirabilis	XDR	BFC 2	PBS	10^7		Topical	200 ml q24h	10 days				Cotrimoxazole (IV) and meropenem (IV)	No	Hospital	No						Yes	Yes	Complete wound healing, patient discharged	SOC MP
20	AbdI	BSI	Acute + chronic	Liver abscesses and sepsis in a liver transplanted patient	P. aeruginosa*	XDR	BFC 1	NaCl 0.9%	10^7	250 ml	IL (biliary drainage catheter)	1ml (infusions in liver absesces) q24h	1 week	IV	22 ml (6h infusion) q24h	86 days	Gentamicin (IV), colistin (IV), and aztreonam (IV)	No	Hospital	Abdominal discomfort	2 days	Mild	Possible	Bacteriophage therapy interrupted	Recovered	Yes	Yes		SOC MP
21	OPI		Chronic	Ewing sarcoma and osteomyelitis of the hip	S. aureus* and Hungatella hathewayi	UDR	BFC 1	PBS	10^7	50 ml	IL (pigtail catheter)	40 ml q8h (week 1) follwed by 40 ml q12h (week 2)	2 weeks				Piperacillin/tazobactam (IV)	No	Hospital	No						Yes	Yes		SOC MP
22	BoneI		Chronic	Chronic pelvic osteomyelitis	M. morganii, P aeruginosa* and Staphylococcus epidermidis*	MDR	BFC 1	PBS	10^7	40 ml**	IL (draining system)	10-20 ml q8h, allowing 10 min. contact time via clampsing**	7 days				Moxifloxacin (oral), vancomycin (IV), and rifampicin (oral)	No	Hospital	No						Yes	Yes	No recurrence of infection	SOC MP
23	BoneI		Chronic	Chronic osteomyelitis of the femur	S. aureus* and Streptococcus agalactiae	UDR	BFC 1	NaCl 0.9%	10^7	40 ml**	IL (draining system)	40 ml q8h, allowing 10 min. contact time via clampsing**	9 days				Vancomycin (IV), clindamycin (oral), and moxifloxacin (oral)	No	Hospital	No						Yes	Yes	No recurrence of infection	SOC MP
24	BoneI		Chronic	Chronic osteomyelitis of the femur	P. aeruginosa* and S. epidermidis*	XDR (P aeruginosa) and UDR (S epidermidis)	BFC 1	PBS	10^7	10-20 ml**	IL (draining system)	10-20 ml q8h, allowing 10 min. contact time via clampsing**	10 days				Colistin (IV), fosfomycin (IV) and vancomycin (IV)	No	Hospital	No						Yes	Yes	No recurrence of infection	SOC MP
25	URTI		Chronic	Chronic rhinosinusitis	Streptococcus pyogenes and S. aureus*	UDR	BFC 1	NaCl 0.9%	10^7		Nasal spray	15 ml q8h	3 weeks					No	Ambulatory	No						Yes	No	Less secretion, better breathing	SOC MP
26	OPI	BSI	Acute + chronic	Dermohypodermatitis of lower limb melanoma and erosive spondylitis with bacteremia	P. aeruginosa*	XDR	4029, 4032, and 4034	NaCl 0.9%	10^6	7 ml	IL (pigtail cathter)	7 ml q24h	3 weeks	IV	30 ml (3h infusion) q24h	3 weeks	Meropenem (IV) and colistin (IV)	No	Hospital	No						Yes	Yes	No pain, perfect scarring, no complications observed on CT scan	ATU MP
27	OPI		Chronic	Chronic osteomyelitis of the femur	P. aeruginosa*	XDR	BFC 1	PBS	10^7	50 ml	IL (pigtail catheter)	40 ml q8h (week 1) followed by 30 ml q12h (week 2)	2 weeks				Linezolid (IV) and ceftazidime/avibactam (IV)	No	Hospital	No						Yes	Yes	Osteomyelitis resolved as observed through MRI	H
28	LRTI		Acute	Lung infection in patient awaiting lung transplantation	A. xylosoxidans*	XDR	APC 2.1	NaCl 0.9%	10^8		Nebulisation	2 ml q6h (day 1) followed by 4 ml q6h (days 2-14)	2 weeks				Piperacillin/tazobactam (IV), tobramycin (IV), meropenem (aerosol), and colistin (aerosol)	No	Hospital	No						No	No		SOC MP
29	LRTI		Chronic	CF lung infection	A. xylosoxidans*	XDR	APC 2.1	NaCl 0.9%	10^8		Nebulisation	4 ml q6h	2 weeks				Azithromycin (IV)	No	Hospital	No						Yes	No		SOC MP
30	URTI		Chronic	Chronic sinusitis	P. aeruginosa* and S aureus*	UDR	BFC 1	NaCl 0.9%	10^7		Nasal spray	15 ml q8h	10 days					No	Ambulatory	No						No	No	Bacteriophage resistance emerged during treatment	SOC MP
31	URTI		Chronic	Chronic sinusitis	S. aureus*	UDR	ISP	NaCl 0.9%	10^7		Nasal spray	15 ml q8h	3 weeks					Yes	Ambulatory	Rash lips	1 day	Moderate	Possible	Bacteriophage therapy stopped	Recovered	Yes	No	Improvement of the sinusal cavities status as observed through fibroscopy	SOC MP
32	URTI		Chronic	Chronic rhinosinusitis	S. aureus*	UDR	ISP	NaCl 0.9%	10^7		Nasal spray	15 ml q8h	3 weeks					No	Ambulatory	No						No	No	Recurrence of symptoms	SOC MP
33	LRTI		Chronic	COPD with bronchiectasis	P. aeruginosa* and Mycobacterium abscessus*	UDR	8UZL and 14-1	NaCl 0.9%	10^8		Nebulisation	2 ml q6h (day 1) followed by 4 ml q6h (days 2-42)	6 weeks					No	Ambulatory	No						No	No	Bacteriophage resistance emerged during treatment	SOC MP
34	SSTI	BSI	Acute + acute	DIC due to pneumococcal sepsis	P. aeruginosa*	XDR	14-1, PNM, and PT07	NaCl 0.9%	10^7		IV	60 ml (6h infusion) q24h	10 days				Colistin (IV) and aztreonam (IV)	No	Hospital	No						Yes	Yes		SOC MP
35	SSTI		Chronic	Burn wounds on both legs	P. aeruginosa* and S. aureus*	UDR	14-1 and ISP	Flaminal® Hydro	10^7		Topical	200 g q12h (covered with Jelonet bandage)	10 days					No	Hospital	No						Yes	No	Burn wound successfully grafted	SOC MP
36	SSTI		Acute	Burn wounds on face, neck, thorax, back, and 4 limbs (TBSA burned: 75%, 2nd and 3rd degree)	P. aeruginosa* and S. aureus*	XDR	PyoPhage	Undiluted	Unknown		Topical	q24h** in excess	1 week				Colistin (IV) and ciprofloxacin (IV)	No	Hospital	No						No	No	Patient deceased due to multi-organ failure	H
37	OPI		Chronic	Chronic prosthetic joint infection of the left shoulder	S. epidermidis*	XDR	ISP	NaCl 0.9%	10^7	40 ml**	IL (pigtail catheter)	20 ml q8h (days 1-5) followed by 20 ml q12h (days 6-10)**	10 days	Topical	Dressings which absorbed the bacteriophage solution	10 days		No	Hospital	No						Yes	Yes	Painless and functional shoulder and no discharge	SOC MP
38	URTI		Chronic	Chronic maxillary sinusitis	P. aeruginosa* and Pseudomonas putida	MDR	14-1, PNM, and PT07	NaCl 0.9%	10^7		Nasal spray	15 ml q8h	3 weeks					No	Ambulatory	No						No	No		SOC MP
39	BoneI		Chronic	Chronic osteomyelitis of the elbow	S. epidermidis*, Enterococcus faecalis, Streptococcus oralis and Citrobacter koseri	UDR	ISP	NaCl 0.9%	10^7	10 ml**	IL (draining system)	10 ml q8h, allowing 10 min. contact time via clampsing**	10 days				Moxyfloxacin (oral) and amoxycillin (oral)	No	Hospital	Fever	1 day	Mild	Possible	None	Recovered	Yes	Yes	The targeted bacterial pathogens (S. epidermidis and E. faecalis were eradicated); another S. epidermidis strain emerged two weeks after bacteriophage therapy	SOC MP
40	BoneI		Chronic	Chronic osteomyelitis of the left sacrum with fistula	S. aureus*, Bacteroides fragilis* and P. aeruginosa*	UDR	14-1, ISP, and UZM3	NaCl 0.9%	10^7		IL (draining system)	20 ml q12h (14-1 and ISP), 20 ml q24h (14-1, ISP and UZM3)	10 days					No	Hospital	No						No	No	Relapse of cancer and the patient died from COVID-19 infection	SOC MP
41	BoneI		Chronic	Chronic osteomyelitis of the tibia with large fistula	P. aeruginosa*, S. aureus*, S. oralis, E. faecalis*	UDR	PNM, ISP, and PyoPhage	NaCl 0.9%, Pyophage undiluted	10^7, Pyophage unknown	2·5 ml**	IL (syringe)	2,5 ml q12h, allowing 15 min. contact time via clampsing**	10 days				Moxyfloxacin (IV and oral) and amoxycillin (IV and oral)	No	Hospital							Yes	No	No pain, fistel reduced in size, normal CRP levels	H
42	BoneI		Chronic	Chronic osteomyelitis of the femur	E. faecalis*	UDR	PyoPhage	Undiluted	Unknown	40 ml**	IL (draining system)	40 ml q8h, allowing 10 min. contact time via clampsing**	1 week				Amoxicillin (IV and oral)	No	Hospital	Local redness and pain	1 day	Moderate	Possible	None	Recovered	Yes	Yes	No recurrence of infection	H
43	AbdI		Acute	Recurrent cholangitis, spleen abscesses, peritonitis and sepsis in liver transplanted patient	E. faecalis, Enterococcus faecium*, Weissella confusa, Enterobacter cloacae and Stenotrophomonas maltophilia	UDR	EFgrKN and EFgrNG	NaCl 0.9%	10^7-10^8		IV	2 ml/kg q12h, infused over 2h (over 4h + 2 mg/kg methylprednisolone on day1); H1-antagonist administered before every bacteriophage application	20 days				Vancomycin (oral)	No	Hospital	No						Yes	No	Recurrent infection, but with a vancomycin susceptible E. faecium strain (the inital strain was vancomycin resistant)	H
44	LRTI	UTI	Acute + chronic	Pulmonary and urinary septic shock	K. pneumoniae*	PDR	M1	NaCl 0.9%	10^7 (bladder), 10^8 (nebulisation)		Nebulisation	2ml q6h (day 1) followed by 4 ml q6h (days 2-14)	2 weeks	Bladder rinsing	20 ml q12h through bladder catheter	10 days	Tigecycline (IV)	Yes	Hospital	Septic shock	NA	Severe	Unlikely	Bacteriophage therapy stopped	Died	No	Yes	Patient died due to a pulmonary septic shock due to A. baumannii; K. pneumoniae no longer isolated from the bladder	H
45	URTI		Chronic	Chronic rhinosinusitis	A. xylosoxidans*	UDR	JWX	NaCl 0.9%	10^7		Nasal spray	15 ml q8h	3 weeks					No	Ambulatory	No						No	No	No improvement after 3 weeks of treatment	H
46	LRTI		Chronic	Disseminated bronchiectasis (lung transplantation considered)	S. aureus*, S. maltophilia*, P. aeruginosa and Scedosporium apiospermum	MDR	ISP and BUCT700	NaCl 0.9%	10^8		Nebulisation	2ml q6h (day 1) followed by 4 ml q6h (days 2-21)	3 weeks					No	Hospital	No						Yes	No	E. coli superinfection	SOC MP
47	LRTI		Chronic	CF lung infection	M. abscessus* and P. aeruginosa	UDR	8UZL	NaCl 0.9%	10^8		Nebulisation	2 ml q6h (day 1) followed by 4 ml q6h (days 2-21)	3 weeks					No	Ambulatory + hospital	No						Yes	No	Slight improvement in lung function during treatment; temporary reduction of microbial load in sputum	SOC MP
48	Other	BSI	Chronic + acute	Infection of TIPS device	P. aeruginosa*	UDR	14-1 and PNM	NaCl 0.9%	10^7		IV	50 ml (6h infusion, sometimes bolus) q24h	4 weeks				Ceftazidime (IV) and ciprofoxacin (IV)	No	Hospital	No						Yes	Yes	Negative blood cultures	SOC MP
49	LRTI		Acute	COVID-19 pneumonia with bacterial superinfection and sepsis	P. aeruginosa*	UDR	14-1 and PT07	NaCl 0.9%	10^8		Nebulisation	2 ml q6h (day 1) followed by 4 ml q6h (days 2-14)	2 weeks				Ceftazidime (IV) and amikacin (IV)	No	Hospital	No						Yes	Yes		SOC MP
50	SSTI		Acute	Burn wounds on the left leg (3rd degree)	P. aeruginosa*	UDR	14-1	NaCl 0.9%	10^7		Topical	250 g q24h (covered with Jelonet bandage)	10 days					No	Hospital	No						Yes	No	Clinical improvement (ready for surgery), but no microbial eradication	SOC MP
51	LRTI		Chronic	COPD with bronchiectasis	P. aeruginosa*	UDR	14-1 and PT07	NaCl 0.9%	10^8		Nebulisation	4 ml q6h	3 weeks					No	Ambulatory	No						No	No	The Patient's condition improved over the first 2 weeks, but then dyspnoe, cough and thick mucous re-appeared	SOC MP
52	BoneI		Chronic	Chronic osteomyelitis and fistula after transfemoral amputation	S. aureus*, P. aeruginosa*, and E. faecalis*	UDR	ISP, EFgrKN, EFgrNG, 14-1, and PNM	NaCl 0.9%	10^7		IL (draining system)	40 ml q8h	10 days				Vancomycin (IV) and ceftazidim (IV)	No	Hospital	No						Yes	Yes		SOC MP
53	LRTI		Chronic	Severe bilateral bronchiectasis	P. aeruginosa*	UDR	PNM	NaCl 0.9%	10^8		Nebulisation	4 ml q6h	6 weeks					No	Ambulatory	No						Yes	Yes	Less exacerbation, but a new bacterial species (K. pneumoniae) emerged post bacteriophage treatment	SOC MP
54	LRTI		Acute	VAP, ARDS due to COVID-19 (intubation, oxygen, mechanical ventilation)	P. aeruginosa*	XDR	14-1, PNM, and PT07	NaCl 0.9%	10^8		Nebulisation	q6h (2 ml day one, 4 ml days 2-7)	1 week				Colistin (IV)	No	Hospital	No						Yes	No	Transferred to a revalidation unit, but no microbial eradication	SOC MP
55	OPI		Chronic	Prosthetic knee infection	S. epidermidis*	UDR	ISP and BE06	NaCl 0.9%	10^7	50 ml (100x diluted) during arthroscopy	IL (draining system)	5 ml q12-24h	3 weeks	IV	50 ml (100x diluted) q24h	1 week		No	Hospital	No						No	No	Recurrent infection with the same S. epidermidis strain	ATU MP
56	Other		Chronic	Persistent LVAD driveline infection	P. aeruginosa*	MDR	14-1, PNM, and PT07	NaCl 0.9%	10^8	50 ml	IV	70 ml (7h infusion) q24h	10 days	Topical	Bacteriophages absorbed in dressings q12h	5 days	Ceftolozane/tazobactam (IV)	No	Hospital	No						Yes	Yes	Infection resolved and wound healed	H
57	LRTI		Chronic	Bronchiectasis	P. aeruginosa*	PDR	14-1 and PNM	NaCl 0.9%	10^8		Nebulisation	2 ml q6h (day 1) followed by 4 ml q6h (days 2-21)	3 weeks				Doxycyclin (oral) and levofloxacin (oral)	No	Ambulatory	No						No	No	No measurable signs of clinical improvement	H
58	SSTI		Chronic	Diabetic foot infection (high risk of amputation)	S. aureus*	UDR	ISP	NaCl 0.9%	10^9		Topical	In excess, up to 4 ml, q24h	6 days				Flucloxacillin (IV) and gentamicin (IV)	Yes	Hospital	Heart failure	NA	Severe	Unlikely	Bacteriophage therapy stopped	Recovered	Yes	Unknown	Clinical effect observed, but bacteriophage therapy stopped after 5 days due to ICU admission upon heart failure	SOC UM
59	SSTI		Chronic	Diabetic foot infection (high risk of amputation)	S. aureus*	UDR	ISP	NaCl 0.9%	10^9		Topical	In excess, up to 4 ml, q24h	16 days				Flucloxacillin (IV)	No	Hospital	No						Yes	Unknown	Excellent clinical response, considered amputation prevented	SOC UM
60	SSTI		Chronic	Diabetic foot infection (high risk of amputation)	S. aureus*	UDR	ISP	NaCl 0.9%	10^9		Topical	In excess, up to 4 ml, q24h	2 weeks				Cotrimoxazole (IV)	No	Hospital	No						Yes	Unknown	Moderate (slow wound healing) clinical response, considered amputation prevented	SOC UM
61	SSTI		Chronic	Diabetic foot infection (high risk of amputation)	S. aureus*	UDR	ISP	NaCl 0.9%	10^9		Topical	In excess, up to 4 ml, q24h	19 days				Gentamicin (IV)	No	Hospital	No						Yes	Unknown	Excellent clinical response, considered amputation prevented	SOC UM
62	SSTI		Chronic	Diabetic foot infection (high risk of amputation)	S. aureus* and Staphylococcus hominis*	UDR	ISP	NaCl 0.9%	10^9		Topical	In excess, up to 4 ml, q24h	2 weeks				Flucloxacillin (IV)	No	Hospital	No						Yes	Unknown	Excellent clinical response, considered amputation prevented	SOC UM
63	SSTI		Chronic	Diabetic foot infection (high risk of amputation)	S. aureus*	UDR	ISP	NaCl 0.9%	10^9		Topical	In excess, up to 4 ml, q24h	2 weeks				Daptomycin (IV) and gentamicin (IV)	No	Hospital	No						No	No	Insignificant clinical response, likely reflecting the low EOP of bacteriophage ISP, and the patient went onto amputation	SOC UM
64	AbdI		Chronic	Chron's disease with anal fistula	P. aeruginosa*	MDR	14-1, PNM, and PT07	PBS	10^7		IL (draining system)	14 ml q12h	2 weeks					No	Ambulatory	No						Yes	Yes	Wounds completely closed	H
65	BoneI		Chronic	Chronic osteomyelitis of the femur	S. aureus*	MDR	ISP	NaCl 0.9%	10^7	20 ml	IL (pigtail catheter)	20 ml q8h	8 days				Teicoplanin (IV) and rifampicin (IV)	No	Hospital	No						Yes	Yes	Complete wound closure	SOC MP
66	LRTI		Chronic	CF lung infection	M. abscessus*	UDR	8UZL	NaCl 0.9%	10^7 (IV), 10^8 (nebulisation)		Nebulisation	2 ml q6h (day 1) followed by 4 ml q6h (days 2-21)	3 weeks	IV	60 ml (6h infusion) q24h	10 days	Amikacin (aerosol), imipenem/cilastine (IV), ceftazidime/avibactam (IV), clofazimine (oral), and minocycline (oral)	No	Ambulatory + hospital	No						No	No		SOC MP
67	LRTI		Acute	Lung infection upon lobectomy	P. aeruginosa*	MDR	PNM	NaCl 0.9%	10^8		Nebulisation	2 ml q6h (day 1) followed by 4 ml q6h (days 2-30)	30 days				Amikacin (IV) and ciprofloxacin (IV)	No	Hospital	No						Yes	Yes	Pneumonia resolved	SOC MP
68	AbdI		Acute	Severe chest and abdominal infection after separation of conjoined twins	K. pneumoniae*	XDR	M1	Undiluted	10^8		IL (draining system, sometimes during VAC, after rinsing with NaCl 0·9%)	12 ml (10x dluted) q24h (days 1-3), followed by 12 ml (5x diluted) q24h (days 4-20)	20 days				Meropenem (IV), colimycin (IV), amikacin (IV), and fluconazole (IV)	No	Hospital	No						Yes	Yes	K. pneumoniae no longer isolated from the wound, while still present in the patient's stool	H
69	AbdI		Acute	Severe chest and abdominal infection after separation of conjoined twins	K. pneumoniae*	XDR	M1	Undiluted	10^8		IL (draining system, sometimes during VAC, after rinsing with NaCl 0·9%)	12 ml (10x dluted) q24h (day 1), followed by 16 ml (5x diluted) q24h (days 2-18)	18 days				Meropenem (IV), colimycin (IV), amikacin (IV), and fluconazole (IV)	Yes	Hospital	Cardiogenic shock	NA	Severe	Unlikely	Bacteriophage therapy stopped	Died	No	No	Patient died due to a cardiogenic shock (left heart hypoplasia)	H
70	LRTI	URTI	Chronic + chronic	Chronic rhinosinusitis and bronchiectasis	S. aureus*	UDR	ISP	NaCl 0.9%	10^7		Nasal spray	15 ml q8h	3 weeks					No	Ambulatory	No						Yes	No	Better breathing, less secretion, and improvement of sinusal cavity inflammation (confirmed by endoscopy), but persistance of P. aeruginosa in the frontal sinus cavity	SOC MP
71	LRTI		Chronic	COPD with bronchiectasis	P. aeruginosa*	UDR	PT07	PBS	10^8		Nebulisation	2 ml q6h (day 1) followed by 4 ml q6h (days 2-21)	6 weeks					No	Ambulatory	No						Yes	Yes		SOC MP
72	SSTI		Chronic	Erysipelas with skin necrosis	S. aureus*	MDR	ISP	Flaminal® Hydro	10^7		Topical	100 g q24h (covered with Jelonet bandage)	1 week				Meropenem (IV)	No	Hospital	No						Yes	Yes	After S. aureus eradication, P. aeruginosa (not sensible to the bacteriophages) emerged in the wound bed	SOC MP
73	SSTI		Chronic	Infection of sacral pressure sores	S. aureus*	UDR	ISP	Flaminal® Hydro	10^7		Topical	100 g q24h (covered with Jelonet bandage)	1 week					No	Hospital	No						Yes	No	Less wound fluid, more red granulation tissue, but no microbial eradication	SOC MP
74	SSTI	BSI	Acute + acute	Electrical burns (TBSA burned: 70%)	P. aeruginosa*	PDR	14-1, PNM, and PT07	NaCl 0.9%	10^7		IV	80 ml (6h infusion) q24h	5 days				Meropenem (IV) and vancomycin (IV)	No	Hospital	No						Yes	Yes		SOC MP
75	LRTI		Acute	VAP (necrotic pneumonia), polytraumatised (car accident)	P. aeruginosa*	MDR	14-1, PNM, and PT07	NaCl 0.9%	10^7 (IV), 10^8 (nebulisation)		Nebulisation	4 ml q6h	6 weeks	IV	70 ml (6h infusion) q24h	10 days	Ceftazidime/avibactam (IV), ciprofloxacine (IV), colistin (IV), and tobramycin (aerosol)	No	Hospital	No						Yes	Yes	No more P. aeruginosa isolated from samples	SOC MP
76	LRTI		Acute	COVID-19 with VAP (VV-ECMO)	P. aeruginosa*	MDR	PNM	PBS	10^8		Nebulisation	2 ml q6h (day 1) followed by 4 ml q6h (days 2-5)	5 days				Ciprofloxacin (IV), amikacin (IV), and colistin (IV)	No	Hospital	No						No	No		SOC MP
77	URTI		Chronic	Chronic rhinosinusitis	S. aureus* and A. xylosoxidans*	MDR	ISP and JWDelta	PBS	10^7		Nasal spray	15 ml q8h	3 weeks					No	Ambulatory	No						Yes	Yes	Less sputum, less fatigue, sleeps better	H
78	URTI		Chronic	Chronic rhinosinusitis	S. aureus*	UDR	ISP	PBS	10^7		Nasal spray	15 ml q8h	3 weeks					No	Ambulatory	No						Yes	Yes	Less sputum, less fatigue, sleeps better	H
79	Other		Chronic	Chronic spondylodiscitis	P. aeruginosa*	XDR	PNM and PT07	NaCl 0.9%	10^8	40 ml	IV	50 ml (2h infusion) q24h	4 days				Cefiderocol (IV)	Yes	Hospital	Postoperative ileus	NA	Severe	Unlikely	Bacteriophage therapy stopped	Died	Yes	No	Fever dropped and immunoprecipitation decreased	H
80	LRTI		Chronic	Primary ciliary dyskinesia with bronchiectasis	P. aeruginosa*	UDR	PT07	Undiluted	10^9		Nebulisation	2 ml q6h (day 1) followed by 4 ml q6h (days 2-42)	6 weeks					No	Ambulatory	No						Yes	No	Fever dropped, no more coughing, but recurrence of coughing 15 days after the end of bacteriophage treatment	H
81	SSTI		Chronic	Persistent LVAD driveline infection	P. aeruginosa*	MDR	PNM and PT07	NaCl 0.9%	10^7		IV	80 ml (6h infusion) q24h	8 days	IL	50 ml q24h**	3 days	Amikacin (IV) and ceftazidime/avibactam (IV)	No	Hospital	No						Yes	Yes	No more inflammation as observed on PET CT scan	H
82	LRTI		Chronic	CF lung infection	P. aeruginosa*	MDR	4P and DP1	NaCl 0.9%	10^8		Nebulisation	2 ml q6h (day 1) followed by 4 ml q6h (days 2-42)	6 weeks				Aztreonam (aerosol), tobramicine (aerosol) and, levofloxacin (oral)	No	Hospital	No						Yes	Yes		H
83	SSTI		Chronic	Diabetic foot infection (high risk of amputation)	S. aureus*	UDR	ISP	NaCl 0.9%	10^9		Topical	In excess, up to 4 ml, q24h	1 week				Vancomycin (IV)	No	Hospital	No						Yes	Unknown	Excellent clinical response, considered amputation prevented	SOC UM
84	SSTI		Chronic	Diabetic foot infection (high risk of amputation)	S. aureus*	UDR	ISP	NaCl 0.9%	10^9		Topical	In excess, up to 4 ml, q24h	15 days				Vancomycin (IV)	No	Hospital	No						Yes	Unknown	Excellent clinical response, considered amputation prevented	SOC UM
85	SSTI		Chronic	Diabetic foot infection (high risk of amputation)	S. aureus* and E. faecalis	UDR	ISP	NaCl 0.9%	10^9		Topical	In excess, up to 4 ml, q24h	8 days				Flucloxacillin (IV)	No	Hospital	No						Yes	Yes	The targeted S aureus strain was eradicated, but an E. faecalis strain turned out to be the primary pathogen	SOC UM
86	SSTI		Chronic	Diabetic foot infection (high risk of amputation)	S. aureus*	MDR	ISP	NaCl 0.9%	10^9		Topical	In excess, up to 4 ml, q24h	23 days				Vancomycin (IV), clindamycin (IV), and piperacillin/tazobactam (IV)	No	Hospital	No						Yes	Yes	Excellent clinical improvement and resolution of infection achieved	SOC UM
87	Other	BSI	Acute + acute	Leriche's syndrome with infected bi-femoral axillo femoral bypass leading to bacteremia	P. aeruginosa*	MDR	14-1, PNM, and PT07	NaCl 0.9%	10^7		IV	70 ml (6h infusion) q24h	1 week				Ceftazidime/avibactam (IV) and amikacin (IV)	No	Hospital	No						No	No	Modified antibiotic resistance profile	H
88	BoneI		Chronic	Chronic prosthetic joint infection of the left knee	E. coli* and E. faecalis*	MDR	E4 and Efs7	NaCl 0.9%	10^8 (IV), 10^9 (intra-articular)	30 ml	IV	50 ml (1 h infusion) q24h	15 days	Intra-articular	3 x 10 ml injections	3 days	Amoxicillin (IV), meropenem (IV), and ertapenem (IV)	No	Hospital	Slight increase in temperature	Transient	Mild	Possible	None	Recovered	Yes	Yes (E faecalis) and No (E coli)	Initially, both E. coli and E. faecalis could no longer be cultured from wound samples. Reinfection of the new prosthesis with E. coli (same strain)	H
89	BoneI		Chronic	Chronic osteomyelitis of the foot	S. aureus*	UDR	ISP	PBS	10^7	20 ml	IL (VAC Ulta™ irrigation and negative pressure system)	10 ml q8h	10 days				Flucloxacillin (IV)	No	Hospital	No						No	No	Amputation	H
90	URTI		Chronic	Chronical sinusitis	S. aureus*	UDR	ISP	NaCl 0.9%	10^7		Nasal spray	15 ml q8h	3 weeks					No	Ambulatory	No						Yes	No	Less mucus, less rhinorrhea, no success mcrobial eradication	H
91	LRTI		Acute	ARDS due to COVID-19, followed by sepsis	P. aeruginosa*	MDR	14-1, PNM and PT07	NaCl 0.9%	10^8 (10^7 for IV)		IV	70 ml q24h (6h infusion)	8 days	Nebulisation	4 ml q6h	10 days	Meropenem (IV), colimycin (IV and aerosol), and vancomycin (IV)	No	Hospital	No						Yes	No (few P aeruginosa still present in ETA)	Resolution of VAP and no more ventilation needed	SOC MP
92	SSTI	LRTI	Acute	Generalised necrotisong fasciitis, empyema, bacterial tracheitis, bacteremia, mitral valve abscess, septic necrosis of left foot	S. aureus*, P. aeruginosa*, S. maltophilia*	MDR	ISP, 14-1, PNM, PT07 and BUCT700	NaCl 0.9%	10^7, 10^8 (nebulisation)	200 ml and soaked dressings, intra-operative on the leg stump before surgical closure (14-1, PNM, PT07, BUCT700)	IV (ISP)	50 ml q24h (6h infusion)	22 days	Generalised bacteriophage therapy: intrapleural (ISP), intraperitoneal (ISP), nebulisation (ISP), topical (ISP)	Intrapleural and intraperitoneal: 20-40 ml q24h**, nebulisation: 2 ml q6h (day 1) followed by 4 ml q6h (days 2-7), topical: in excess	1 week	Vancomycin (IV), clindamycin (IV), and ceftaroline (IV)	No	Hospital	No						Yes	Yes	Favorable clinical progression along durable eradication of all S. aureus, P. aeruginosa, and S. maltophilia and from the bloodstream and all wounds. Full recovery of all wounds and of respiratory function.	SOC MP
93	LRTI		Acute	Empyema and spinocellular carcinoma	P. aeruginosa*, K. pneumoniae, and Actinomyces spp.	MDR	14-1	NaCl 0.9%	10^7 (thorax drain) 10^8 (nebulization)		IL (thorax drain)	50 ml q8h (30 min infusion)	7 days	Nebulization	2 ml q4h (day 1) followed by 4 ml q4h (days 2-7) followed by 4 ml q6h (days 8-14)	14 days	Colistin (IV), ceftolozane/tazobactam (IV) and levofloxacine (oral)	Yes	Hospital	Tumor progression	NA	Severe	Unlikely	Bacteriophage therapy stopped	Died	No	Yes	Patient died due to tumor progression (comfort treatment)	SOC MP
94	AbdI	BSI	Acute	Enteric polyp resection, necrotizing hemorrhagic acute pancreatitis, laprotomy and septic shock	E. faecium*, K. pnuemoniae, and C. perfringens	MDR	KN	NaCl 0.9%	10^7		IV	60 ml q24h (days 1-2) followed by 120 ml q24h (6 h infusion).	12 days	IL (Intra-abdominal instillations trhough drainage catheter)	60 ml q24h	12 days	Vancomycin (IV) and Linezolid (IV)	No	Hospital	No						Yes	Yes		SOC MP
95	SSTI		Acute	Echtyma gangrenosum in a liver transplant patient	P. aeruginosa*	UDR	4P and Phage C	NaCl 0.9%	10^7	100 ml	Topical	Dressings soaked in phagees applied on the wound site for 20 min q48h	7 days				Piperacillin/tazobactam(IV) and ciprofloxacin (IV)	No	Hospital	No						Yes	Yes		SOC MP
96	SSTI	BSI	Acute	Burn wounds and septic shock	P. aeruginosa* and E. cloacae	MDR	14-1, PNM and PT07	NaCl 0.9%	10^7		Topical	In excess	1 day	IV (only 14-1)	90 ml q6h (6 h infusion)	5 days	Ceftazidime (IV) and meropenem (IV)	Yes	Hospital	Septic shock	NA	Severe	Unlikely	Bacteriophage therapy stopped	Died	No	No	Patient died during hage therapy, due to septic shock	SOC MP
97	BoneI		Chronic	Osteomyelitis of trochanter with fistula	S. aureus* and M. catharalis	UDR	ISP	NaCl 0.9%	10^7	40 ml	Topical	40 ml**	14 days				Flucloxacillin (IV), piperacillin/tazobactam (IV), and meropenem (IV)	No	Hospital	No						Yes	Yes		SOC MP
98	Other	BSI	Acute	Septic polyarthritis and bacteremia	S. aureus*	UDR	ISP	NaCl 0.9%	10^7		IV	132 ml q24h (6 h infusion)	7 days				Flucloxacillin (IV)	No	Hospital	No						Yes	Yes		SOC MP
99	URTI		Chronic	Chronical sinusitis	S. aureus*	UDR	ISP	NaCl 0.9%	10^7		Nasal spray	15 ml q8h	3 weeks				Doxycycline (oral) and amoxicillin/clavulanic acid (oral)	No	Ambulatory	Diarrhea and abdominal pain	25 days	Moderate	Possible	Medication (amoxiccilin/clavulanic acid)	Recovered	Yes	Unknown		H
100	SSTI		Chronic	subcutaneous surgical wound infection (superficial collection and fistula)	S. aureus*	UDR	ISP	NaCl 0.9%	10^7	200 ml	IL (draining system)	10 ml q8h**	7 days				Clindamycin (IV and oral) and cefazolin (IV)	No	Hospital	Nausea	1 day	Mild	Unlikely (had surgery)	Medication (ondansetron)	Recovered	Yes	Yes	S. aureus no longer isolated	SOC MP

*Targeted by bacteriophage therapy

**Rinsed with 1-3% bicarbonate prior to bacteriophage application

AbdI, abdominal infection; ARDS, acute respiratory distress syndrome; ATU, autorisation temporaire d'utilisation; BoneI, bone infection; BSI, bloodstream infection; CF, cystic fibrosis; COPD, chronic obstructive pulmonary disease; COVID, coronavirus disease; CRP, C-reactive protein; CT, computerised tomography; DIC, disseminated intravascular coagulation; ECMO, extracorporeal membrane oxygenation; EOP, efficiency of plating; ETA, endotracheal aspiration; H, Article 37 of the Declaration of Helsinki; IL, intralesional; ICU, intensive care unit; IV, intravenous; LRTI, lower respiratory tract infection; LVAD, left ventricular assist device; MDR, multidrug resistant; MP, magistral preparation; MRI, magnetic resonance imaging; OPI, orthopedic prostheses infection; PBS, phosphate buffered saline; PDR, pandrug resistant; PET, positron emission tomography; PFU, plaque forming units; SOC, standard of care (magistral preparation); SSTI, skin and soft tissue infection; TBSA, total body surface area; TIPS, transjugular intrahepatic portosystemic shunt; UDR, usual drug resistance; UM, unlicensed medicine; URTI, upper respiratory tract infection; UTI, urinary tract infection; VAC, vacuum-assisted closure; VAP, ventilator-associated pneumonia; VV-ECMO, venovenous extracorporeal membrane oxygenation; XDR, extensively drug resistant.

Supplementary Table 2 | Overview of the first 100 consecutive bacteriophage therapy cases facilitated by a Belgian consortium, encoded, and with eradication predictions added

CASENUM	INDICATI	TARGET	OTHERSP	ABRPROF	APPROUTE	ABCONCOM	CLINSETT	ADVREACT	PTABORT	CI	ERADIC	PREDICTED ERADIC
1	BoneI	SA	Yes	MDR	IL	Yes	H	No	No	Yes	Yes	yes
2	BoneI	SA	No	UDR	IL	Yes	H	No	No	No	No	yes
3	LRTI	PA	Yes	MDR	Nebul	Yes	H	No	Yes	No	No	yes
4	BoneI	PA	No	MDR	Topical	Yes	H	No	No	Yes	Yes	yes
5	Other	EC	No	MDR	Rectal	Yes	A	No	No	Yes	Yes	yes
6	LRTI	EC+PM	No	MDR	Nebul	No	H	No	No	Yes	Yes	No
7	URTI	PA	No	UDR	Nasal	No	A	No	No	Yes	Yes	No
8	LRTI+UTI	PA	No	MDR	Nebul	Yes	H	No	No	Yes	Yes	yes
9	BoneI	KP	No	MDR	IL	Yes	H	No	No	Yes	Yes	yes
10	URTI	SA	No	UDR	Nasal	No	A	No	No	Yes	Yes	No
11	LRTI	PA	No	MDR	Nebul+Oral	No	H	Yes	No	Yes	No	No
12	LRTI	PA	No	UDR	Nebul	No	H	No	No	Yes	Yes	No
13	SSTI+BSI	PA	No	MDR	IV	No	H	No	No	Yes	Yes	No
14	SSTI	PA+SA	No	MDR	Topical	No	H	No	No	No	No	No
15	OPI	PA	No	UDR	IL	Yes	H	No	No	Yes	Yes	yes
16	LRTI	AX	No	MDR	Nebul+IL	No	H	No	No	Yes	Yes	No
17	LRTI	PA	No	MDR	Nebul+Oral	Yes	H	No	No	Yes	Yes	yes
18	SSTI	PA	No	MDR	Topical	No	H	No	No	Yes	Yes	No
19	SSTI	AB	Yes	MDR	Topical	Yes	H	No	No	Yes	Yes	yes
20	AbdI+BSI	PA	No	MDR	IL+IV	Yes	H	Yes	No	Yes	Yes	yes
21	OPI	SA	Yes	UDR	IL	Yes	H	No	No	Yes	Yes	yes
22	BoneI	PA+SE	Yes	MDR	IL	Yes	H	No	No	Yes	Yes	yes
23	BoneI	SA	Yes	UDR	IL	Yes	H	No	No	Yes	Yes	yes
24	BoneI	PA+SE	No	MDR	IL	Yes	H	No	No	Yes	Yes	yes
25	URTI	SA	Yes	UDR	Nasal	No	A	No	No	Yes	No	No
26	OPI+BSI	PA	No	MDR	IL+IV	Yes	H	No	No	Yes	Yes	yes
27	OPI	PA	No	MDR	IL	Yes	H	No	No	Yes	Yes	yes
28	LRTI	AX	No	MDR	Nebul	Yes	H	No	No	No	No	yes
29	LRTI	AX	No	MDR	Nebul	Yes	H	No	No	Yes	No	yes
30	URTI	PA+SA	No	UDR	Nasal	No	A	No	No	No	No	No
31	URTI	SA	No	UDR	Nasal	No	A	Yes	Yes	Yes	No	No
32	URTI	SA	No	UDR	Nasal	No	A	No	No	No	No	No
33	LRTI	PA+MA	No	UDR	Nebul	No	A	No	No	No	No	No
34	SSTI+BSI	PA	No	MDR	IV	Yes	H	No	No	Yes	Yes	yes
35	SSTI	PA+SA	No	UDR	Topical	No	H	No	No	Yes	No	No
36	SSTI	PA+SA	No	MDR	Topical	Yes	H	No	No	No	No	yes
37	OPI	SE	No	MDR	IL+Topical	No	H	No	No	Yes	Yes	No
38	URTI	PA	Yes	MDR	Nasal	No	A	No	No	No	No	No
39	BoneI	SE	Yes	UDR	IL	Yes	H	Yes	No	Yes	Yes	yes
40	BoneI	PA+SA+BF	No	UDR	IL	No	H	No	No	No	No	No
41	BoneI	PA+SA+EF	Yes	UDR	IL	Yes	H	No	No	Yes	No	yes
42	BoneI	EF	No	UDR	IL	Yes	H	Yes	No	Yes	Yes	yes
43	AbdI	EFUM	Yes	UDR	IV	Yes	H	No	No	Yes	No	yes
44	LRTI+UTI	KP	No	MDR	Nebul+BI	Yes	H	No	Yes	No	Yes	yes
45	URTI	AX	No	UDR	Nasal	No	A	No	No	No	No	No
46	LRTI	SA+SM	Yes	MDR	Nebul	No	H	No	No	Yes	No	No
47	LRTI	MA	Yes	UDR	Nebul	No	H+A	No	No	Yes	No	No
48	Other+BSI	PA	No	UDR	IV	Yes	H	No	No	Yes	Yes	yes
49	LRTI	PA	No	UDR	Nebul	Yes	H	No	No	Yes	Yes	yes
50	SSTI	PA	No	UDR	Topical	No	H	No	No	Yes	No	No
51	LRTI	PA	No	UDR	Nebul	No	A	No	No	No	No	No
52	BoneI	PA+SA+EF	No	UDR	IL	Yes	H	No	No	Yes	Yes	yes
53	LRTI	PA	No	UDR	Nebul	No	A	No	No	Yes	Yes	No
54	LRTI	PA	No	MDR	Nebul	Yes	H	No	No	Yes	No	yes
55	OPI	SE	No	UDR	IL+IV	No	H	No	No	No	No	No
56	Other	PA	No	MDR	Topical	Yes	H	No	No	Yes	Yes	yes
57	LRTI	PA	No	MDR	Nebul	Yes	A	No	No	No	No	yes
58	SSTI	SA	No	UDR	Topical	Yes	H	No	Yes	Yes		yes
59	SSTI	SA	No	UDR	Topical	Yes	H	No	No	Yes		yes
60	SSTI	SA	No	UDR	Topical	Yes	H	No	No	Yes		yes
61	SSTI	SA	No	UDR	Topical	Yes	H	No	No	Yes		yes
62	SSTI	SA+SHOM	No	UDR	Topical	Yes	H	No	No	Yes		yes
63	SSTI	SA	No	UDR	Topical	Yes	H	No	No	No	No	yes
64	AbdI	PA	No	MDR	IL	No	A	No	No	Yes	Yes	No
65	BoneI	SA	No	MDR	IL	Yes	H	No	No	Yes	Yes	yes
66	LRTI	MA	No	UDR	Nebul+IV	Yes	H+A	No	No	No	No	yes
67	LRTI	PA	No	MDR	Nebul	Yes	H	No	No	Yes	Yes	yes
68	AbdI	KP	No	MDR	IL	Yes	H	No	No	Yes	Yes	yes
69	AbdI	KP	No	MDR	IL	Yes	H	No	Yes	No	No	yes
70	LRTI+URTI	SA	No	UDR	Nasal	No	A	No	No	Yes	No	No
71	LRTI	PA	No	UDR	Nebul	No	A	No	No	Yes	Yes	No
72	SSTI	SA	No	MDR	Topical	Yes	H	No	No	Yes	Yes	yes
73	SSTI	SA	No	UDR	Topical	No	H	No	No	Yes	No	No
74	SSTI+BSI	PA	No	MDR	IV	Yes	H	No	No	Yes	Yes	yes
75	LRTI	PA	No	MDR	Nebul+IV	Yes	H	No	No	Yes	Yes	yes
76	LRTI	PA	No	MDR	Nebul	Yes	H	No	No	No	No	yes
77	URTI	SA+AX	No	MDR	Nasal	No	A	No	No	Yes	Yes	No
78	URTI	SA	No	UDR	Nasal	No	A	No	No	Yes	Yes	No
79	Other	PA	No	MDR	IV	Yes	H	No	Yes	Yes	No	yes
80	LRTI	PA	No	UDR	Nebul	No	A	No	No	Yes	No	No
81	SSTI	PA	No	MDR	IV	Yes	H	No	No	Yes	Yes	yes
82	LRTI	PA	No	MDR	Nebul	Yes	H	No	No	Yes	Yes	yes
83	SSTI	SA	No	UDR	Topical	Yes	H	No	No	Yes		yes
84	SSTI	SA	No	UDR	Topical	Yes	H	No	No	Yes		yes
85	SSTI	SA	Yes	UDR	Topical	Yes	H	No	No	Yes	Yes	yes
86	SSTI	SA	No	MDR	Topical	Yes	H	No	No	Yes	Yes	yes
87	Other+BSI	PA	No	MDR	IV	Yes	H	No	No	No	No	yes
88	BoneI	EC+EF	No	MDR	IL+IV	Yes	H	Yes	No	Yes	No	yes
89	BoneI	SA	No	UDR	IL	Yes	H	No	No	No	No	yes
90	URTI	SA	No	UDR	Nasal	No	A	No	No	Yes	No	No
91	LRTI	PA	No	MDR	Nebul+IV	Yes	H	No	No	Yes	No	yes
92	SSTI+LRTI	SA+PA+SM	No	MDR	IL+Topical+IV+Nebul	Yes	H	No	No	Yes	Yes	yes
93	LRTI	PA	Yes	MDR	Nebul+IL	Yes	H	No	Yes	No	Yes	yes
94	AbdI+BSI	EFUM	Yes	MDR	IL+IV	Yes	H	No	No	Yes	Yes	yes
95	SSTI	PA	No	UDR	Topical	Yes	H	No	No	Yes	Yes	yes
96	SSTI+BSI	PA	Yes	MDR	Topical	Yes	H	No	Yes	No	No	yes
97	BoneI	SA	Yes	UDR	Topical	Yes	H	No	No	Yes	Yes	yes
98	Other+BSI	SA	No	UDR	IV	Yes	H	No	No	Yes	Yes	yes
99	URTI	SA	No	UDR	Nasal	Yes	A	Yes	No	Yes		yes
100	SSTI	SA	No	UDR	IL	Yes	H	No	No	Yes	Yes	yes

A, ambulatory; AbdI, abdominal infection; AB, *Acinetobacter baumannii*; AX, *Achromobacter xylosoxidans*; BF, *Bacteroides fragilis*; BI, bladder injection; BoneI, bone infection; BSI, bloodstream infection; EC, *Escherichia coli*; EF, *Enterococcus faecalis;* EFUM, *Enterococcus faecium*; H, hospital; IL, intralesional; IV, intravenous; KP, *Klebsiella pneumoniae*; LRTI, lower respiratory tract infection; MA, *Mycobacterium abscessus*; MDR, multidrug resistant; Nebul, nebulization; OPI, orthopedic prostheses infection; PA, *Pseudomonas aeruginosa*; PM, *Proteus mirabilis*; SA, *Staphylococcus aureus*; SE, *Staphylococcus epidermidis*; SHOM, *Staphylococcus hominis*; SM, *Stenotrophomonas maltophilia*; SSTI, skin and soft tissue infection; UDR, usual drug resistance; URTI, upper respiratory tract infection; UTI, urinary tract infection.

Variable	Description
**CASENUM**	Patient case number
**INDICATI**	Infection type
**TARGET**	Bacterial species targeted by bacteriophage therapy
**OTHERSP**	Other species present (not targeted)
**ABRPROF**	Antibiotics resistance profile
**APPROUTE**	Application route
**ABCONCOM**	Concomittant standard of care antibiotics treatment
**CLINSETT**	Ambulatory or hospitalized
**ADVREACT**	Reported adverse reaction
**PTABORT**	Bacteriophage therapy aborted
**CI**	Clinical improvement
**ERADIC**	Eradication of the targeted bacteria
**PREDICTED ERADIC**	Predicted eradication (green fill color for correct prediction)

Supplementary Table 3 | Characteristics of the 26 bacteriophages used, individually or in combination, in the present 100 consecutive bacteriophage therapy cases

Bacterial host species	Name	Classification (family, genus)	Morphotype	Genome accession #	Genome size (kb)	Predicted lifestyle	Origin	Source	Propagation strain used in production	# patients treated
Pseudomonas aeruginosa	14-1	*Caudoviricetes, Pbunavirus*	Myovirus	NC_011703	66.2	SL	SIGSIM and KU Leuven	Sewage water, Regensburg, 2000	573	22
	PNM	*Autographiviridae, Phikmvirus*	Podovirus	OP292288	42.7	SL	EIBMV	Mtkvari River, Tbilisi, 1999	573	21
	PT07	*Caudoviricetes, Pakpunavirus*	Myovirus	OQ850183	94.7	SL	EIBMV	Lake Ku, Tbilisi, 1999	573	18
	4029	*Schitoviridae, Litunavirus*	Podovirus	ON815901	72.0	SL	University of Lausanne	Sewage water, Lausanne, 2015	PAO1	1
	4032	*Schitoviridae, Litunavirus*	Podovirus	ON815902	72.0	SL	University of Lausanne	Sewage water, Lausanne, 2015	ATCC15442	1
	4034	*Schitoviridae, Litunavirus*	Podovirus	ON815903	72.0	SL	University of Lausanne	Sewage water, Lausanne, 2015	PAO1	1
	4P	*Caudoviricetes, Pbunavirus*	Myovirus	OQ872152	66.1	SL	QAMH	Congo Stream, Congo, 2016	573	2
	DP1	*Caudoviricetes, Pbunavirus* genus	Myovirus	NC_041870	66.2	SL	University of Minho	Hospital waste water, Portugal, 2010	573	1
	Phage C	*Caudoviricetes, Pbunavirus*	Myovirus	OQ925957	65.6	SL	QAMH	Hospital waste water, Belgium, 2020	573	1
Achromobacter xylosoxidans	JWAlpha	*Schitoviridae, Jwalphavirus*	Podovirus	NC_023556	72.3	SL	DSMZ	Treatment plant waste water, Germany, 2012	1215103	0*
	JWDelta	*Schitoviridae, Jwalphavirus*	Podovirus	KF787094	73.7	SL	DSMZ	Treatment plant waste water, Germany, 2012	1215103	1
	JWT	*Caudoviricetes, Steinhofvirus*	Siphovirus	OQ938574	50.0	SL	DSMZ	Treatment plant waste water, Germany, 2012	1215103	0*
	2-1	*Caudoviricetes*	Siphovirus	OQ974181	82.7	SL	DSMZ	Treatment plant waste water, Germany, 2012	1215103	0*
	JWX	*Caudoviricetes, Steinhofvirus*	Siphovirus	NC_028768	49.7	SL	DSMZ	Treatment plant waste water, Germany 2012	ATCC27061	1
Acinetobacter baumannii	Acibel004	*Caudoviricetes Saclayvirus*	Myovirus	NC_025462	99.7	SL	QAMH	Hospital waste water, Ghent, 2009	070517/0072	0*
	Acibel007	*Autographiviridae, Daemvirus*	Podovirus	NC_025457	42.7	SL	QAMH	Hospital waste water, Ghent, 2009	070517/0072	0*
Enterococus spp.	EFGrKN	*Herelleviridae, Schiekvirus*	Myovirus	MW004544	147.5	SL	Hebrew University, Jerusalem	Sewage water, Jerusalem, 2019	GE1555-19	3
	EFGrNG	*Herelleviridae, Schiekvirus*	Myovirus	MW004545	145.2	SL	Hebrew University, Jerusalem	Sewage water, Jerusalem, 2019	GE1555-19	2
	Efs7	*Caudoviricetes, Saphexavirus*	Siphovirus	OL870612	56.1	SL	ETH Zürich	Waste water, Switzerland, 2020	EF57	1
Bacteroides fragilis	UZM3	*Caudoviricetes*	Siphovirus	OQ116603	46.0	SL	QAMH	Hospital waste water, Ghent, 2010	V-191118-109	1
Escherichia coli	E4	*Straboviridae, Tequatrovirus*	Myovirus	OL870317	169.2	SL	ETH Zurich	Waste water, Switzerland, 2020	BL-21	1
Klebsiella pneumoniae	M1	*Straboviridae, Slopekvirus*	Myovirus	MW448170	176.3	SL	EIBMV	Sewage water, Tbilisi, 2012	Kp040762	4

Mycobacterium abscessus	8UZL	*Caudoviricetes, Mapvirus*	Siphovirus	OQ988004	47.8	SL	QAMH	Hospital waste water, Leuven, 2016	25300801D	3
Stenotrophomonas maltophilia	BUCT700	*Autographiviridae, Phikmvirus*	Podovirus	OM735686	43.2	SL	BUCT	Hospital waste water, Beijing, 2012	19008140792	2
Staphylococcus aureus	ISP	*Herelleviridae, Kayvirus*	Myovirus	NC_047720	138.3	SL	EIBMV	Unknown, Tbilisi, 1920-1930	13S44S	33
Staphylococcus epidermidis	BE06	*Herelleviridae, Sepunavirus*	Myovirus	MT596503	140.7	SL	University of Bern	Human skin, Bern, 2019	SKNA49	1

BUCT, Beijing University of Chemical Technology; DSMZ, "Deutsche Sammlung von Mikroorganismen und Zellkulturen" (German Collection of Microorganisms and Cell Cultures); EIBMV, Eliava Institute of Bacteriophages (Tbilisi), Microbiology and Virology; ETH Zürich, Eidgenössische Technische Hochschule Zürich; KU Leuven, Katholieke Universiteit Leuven; QAMH, Queen Astrid military hospital (Brussels); SIGSIM, State Institute for Genetics and Selection of Industrial Microorganisms (Moscow); SL, strictly lytic. *Always applied as part of a bacteriophage cocktail (see Extended Data Table 2).

Supplementary Table 4 | Characteristics of the six defined bacteriophage cocktails used in the present 100 consecutive bacteriophage therapy cases

Name	Bacterial host species	# bacteriophages	Bacteriophage names	Origin	# patients treated
BFC 1	*Pseudomonas aeruginosa* and *Staphylococcus aureus*	3	14-1, PNM, and ISP	QAMH	14
BFC 2	*Acinetobacter baumannii*, *P. aeruginosa*, and *S. aureus*	5	Acibel004, Acibel007, 14-1, PNM, and ISP	QAMH	8
PyoPhage	*Enterococcus* (*faecalis* and *faecium*), *Escherichia coli*, *Proteus mirabilis*, *P. aeruginosa*, and *S. aureus*	18	Bacteriophages are not named	EIBMV	3
IntestiPhage	*Shigella* (*flexneri*, *sonnei*, Newcastle), *Salmonella* (Paratyphi A, Paratyphi B, Typhumurium, Enteritidis, Cholerasuis, Oranienburg), *E. coli*, *P. vulgaris* and *mirabilis*, *S. aureus*, *P. aeruginosa* and *Enterococcus* spp.	23 “bacteriophage clusters”	Bacteriophages are not named	EIBMV	1
APC 1.1	*Achromobacter xylosoxidans*	1	JWAlpha, JWDelta, and JWT	DSMZ	1
APC 2.1	*A. xylosoxidans*	3	JWAlpha, JWDelta, JWT, and 2-1	DSMZ	3

BUCT, Beijing University of Chemical Technology; DSMZ, “Deutsche Sammlung von Mikroorganismen und Zellkulturen” (German Collection of Microorganisms and Cell Cultures); EIBMV, Eliava Institute of Bacteriophages, Microbiology and Virology (Tbilisi); QAMH, Queen Astrid military hospital (Brussels).
