# Supplementary material for: Personalized bacteriophage therapy outcomes for 100 consecutive cases: a multicentre, multinational, retrospective observational study
Source: Nat Microbiol. 2024 Jun 4;9(6):1434–53. doi: 10.1038/s41564-024-01705-x (PMC11153159; doi:10.1038/s41564-024-01705-x)
Supplement: Supplementary file 2 — Reporting Summary [file 41564_2024_1705_MOESM2_ESM.pdf]

nature portfolio

Corresponding author(s): Jean-Paul Pirnay
Last updated by author(s): March 27, 2024

Reporting Summary

Nature Portfolio wishes to improve the reproducibility of the work that we publish. This form provides structure for consistency and transparency in reporting. For further information on Nature Portfolio policies, see our Editorial Policies and the Editorial Policy Checklist.

Statistics

For all statistical analyses, confirm that the following items are present in the figure legend, table legend, main text, or Methods section.

n/a	Confirmed	
☐	☒	The exact sample size (n) for each experimental group/condition, given as a discrete number and unit of measurement
☐	☒	A statement on whether measurements were taken from distinct samples or whether the same sample was measured repeatedly
☐	☒	The statistical test(s) used AND whether they are one- or two-sided<br>*Only common tests should be described solely by name; describe more complex techniques in the Methods section.*
☐	☒	A description of all covariates tested
☐	☒	A description of any assumptions or corrections, such as tests of normality and adjustment for multiple comparisons
☐	☒	A full description of the statistical parameters including central tendency (e.g. means) or other basic estimates (e.g. regression coefficient) AND variation (e.g. standard deviation) or associated estimates of uncertainty (e.g. confidence intervals)
☐	☒	For null hypothesis testing, the test statistic (e.g. F, t, r) with confidence intervals, effect sizes, degrees of freedom and P value noted<br>*Give P values as exact values whenever suitable.*
☒	☐	For Bayesian analysis, information on the choice of priors and Markov chain Monte Carlo settings
☒	☐	For hierarchical and complex designs, identification of the appropriate level for tests and full reporting of outcomes
☒	☐	Estimates of effect sizes (e.g. Cohen's d, Pearson's r), indicating how they were calculated

Our web collection on statistics for biologists contains articles on many of the points above.

Software and code

Policy information about availability of computer code

Data collection	REDCap (Research Electronic Data Capture) and Microsoft Excel (version 16.77)
Data analysis	Trimmomatic (version 0.32), Guppy (versions 6.0.1 and 6.3.8), Unicycler (versions 0.4.7 and 0.4.8), SPAdes (Galaxy Version 3.15.4+), Prokka (version Galaxy 1.14.6), PHASTER (https://phaster.ca/), Prophage Hunter (https://pro-hunter.bgi.com/), Biolog Data Analysis software (version 1.7), Porechop (version 0.2.4), Snippy (version 4.6.0), EggNOG-mapper (version 2.1.8), mobileOG-db (version 1.1.2), Phigaro (version 2.3.0), circos (version 0.69.8), and GC-profile, Roary (version 3.13.0), fasttree (version 2.1.10), iTOL (itol.embl.de), SAS (version 9.4), R (version 4.3.0), tidyverse (version 2.0.0), UpSetR (version 1.4.0), ggmap (version 3.0.2), rnaturalearth (R package version 0.3.2.9000), and GraphPad V (version 0.5.1), the PHROGS v3 database (https://phrogs.lmge.uca.fr/)ARG-ANNOT (ARG-ANNOT NT V6 July 2019), CARD (Versions 3.1.4 to 3.2.5), ResFinder (https://bitbucket.org/genomicepidemiology/resfinder_db), blastn web interface (https://blast.ncbi.nlm.nih.gov/Blast.cgi), VFDB full (downloaded on 20 April 2022), Bowtie2 (Galaxy Version 2.5.0)

For manuscripts utilizing custom algorithms or software that are central to the research but not yet described in published literature, software must be made available to editors and reviewers. We strongly encourage code deposition in a community repository (e.g. GitHub). See the Nature Portfolio guidelines for submitting code & software for further information.

Data

Policy information about availability of data

All manuscripts must include a data availability statement. This statement should provide the following information, where applicable:

- Accession codes, unique identifiers, or web links for publicly available datasets
- A description of any restrictions on data availability
- For clinical datasets or third party data, please ensure that the statement adheres to our policy

Detailed clinical protocols, results, and additional data are available in the manuscript and in Supplementary Tables 1 and 2. The protocol for the retrospective, observational study is available at: https://clinicaltrials.gov/ct2/show/NCT05498363?term=NCT05498363&draw=2&rank=l. The bacteriophage genome sequences can be retrieved in the GenBank database under the accession codes listed in Extended Data Table 1. The genome data of the bacterial isolates can be accessed via the NCBI BioProject PRJNA975428. PHROGS v3 database available at https://phrogs.lmge.uca.fr/. The authors declare that all other data supporting the findings of this study are available within the article.

Research involving human participants, their data, or biological material

Policy information about studies with human participants or human data. See also policy information about sex, gender (identity/presentation), and sexual orientation and race, ethnicity and racism.

Reporting on sex and gender	The median age of the patients was 53 years (1–91 years), and 56.7% of the patients were male. No effects of age or gender on eradication were found using a logistic regression analysis.
Reporting on race, ethnicity, or other socially relevant groupings	Race, ethnicity, or other socially relevant groupings were not considered or reported.
Population characteristics	The median age of the patients was 53 years (1–91 years), and 56.7% of the patients were male. No effects of age or gender on eradication were found using a logistic regression analysis.
Recruitment	This study concerns the first 100 consecutive bacteriophage therapy (BT) cases facilitated by a Belgian consortium (single group, open label). Physicians requesting BT with bacteriophage preparations for their patients submitted a BT request to the Phage Therapy Coordination Centre (PTCC) of the QAMH. The PTCC procedure for selecting patients for BT is depicted in Extended Data Figure 1 and is largely determined by clinical need, regulatory approval, and the availability of bacteriophages targeting the infecting bacteria.
Ethics oversight	According to EU Regulation No 536/2014 (Clinical Trials Regulation), its transposition to Belgian Law, and per advice of the Leading Ethical Committee of the "Universitair Ziekenhuis Antwerpen" and the "Universiteit Antwerpen" (ID 3644), which approved the observational study protocol. The present retrospective non-interventional analysis of an existing and de-identified BT database was not considered as an experiment on the human person and did not require a dedicated informed consent. This information is also provided in the manuscript.

Note that full information on the approval of the study protocol must also be provided in the manuscript.

Field-specific reporting

Please select the one below that is the best fit for your research. If you are not sure, read the appropriate sections before making your selection.

☒ Life sciences ☐ Behavioural & social sciences ☐ Ecological, evolutionary & environmental sciences

For a reference copy of the document with all sections, see nature.com/documents/nr-reporting-summary-flat.pdf

Life sciences study design

All studies must disclose on these points even when the disclosure is negative.

Sample size	100 patients (retrospective report no sample predetermined). For *Galleria mellonella* sample size was based on previous publication Nieuwenhuyse et al., 2022)
Data exclusions	For Galleria model one experiment was excluded from the analysis as different time points and measurements were taken.
Replication	Bacteriophage - antibiotic synergy testing was performed only once, in an emergency routine setting, prior to the application of bacteriophages. For Galleria model this experiment was repeated once. All other experiments were repeated at least 3 times.
Randomization	Patients were not randomised. The study has a single group design. For *G. mellonella,* larvae were randomly assigned.
Blinding	There was no blinding. The study has an open label design. For *G. mellonella* the assessment was not blinded.

Reporting for specific materials, systems and methods

We require information from authors about some types of materials, experimental systems and methods used in many studies. Here, indicate whether each material, system or method listed is relevant to your study. If you are not sure if a list item applies to your research, read the appropriate section before selecting a response.

Materials & experimental systems

n/a	Involved in the study	
☒	☐	Antibodies
☒	☐	Eukaryotic cell lines
☒	☐	Palaeontology and archaeology
☐	☒	Animals and other organisms
☐	☒	Clinical data
☒	☐	Dual use research of concern
☒	☐	Plants

Methods

n/a	Involved in the study	
☒	☐	ChIP-seq
☒	☐	Flow cytometry
☒	☐	MRI-based neuroimaging

Animals and other research organisms

Policy information about studies involving animals; ARRIVE guidelines recommended for reporting animal research, and Sex and Gender in Research

Laboratory animals	Species is Galleria mellonella; strain is unspecified; sex indifferent; stage larvae; age about 3 weeks
Wild animals	No wild animals used in this study
Reporting on sex	Sex was not considered
Field-collected samples	The study did not involve samples collected from the field
Ethics oversight	It was considered that use of this invertebrate model did not require specific guidance.

Note that full information on the approval of the study protocol must also be provided in the manuscript.

Clinical data

Policy information about clinical studies

All manuscripts should comply with the ICMJE guidelines for publication of clinical research and a completed CONSORT checklist must be included with all submissions.

Clinical trial registration	Study BT100, ID: NCT05498363
Study protocol	The protocol for the retrospective, observational study is available at: https://clinicaltrials.gov/ct2/show/NCT0S498363?term=NCT0S498363&draw=2&rank=l.
Data collection	Prior to BT, demographic and clinical data were collected through a medical form, which was completed by the Bacteriophage Therapy Providers. The medical doctor's BT prescription, information regarding the applied bacteriophage product and its administration route, dosage, duration, and information with regard to possible concomitant (antibiotic) treatments were also recorded. The "phagograms", reporting on the evaluation of the bacteriophage susceptibility of the patient's bacterial isolates sampled before and sometimes during treatment were also archived. If the bacteriophage treatment was performed in a hospital, a clinical follow-up form, requesting information about the clinical outcome (incl. possible adverse events and reactions), was completed by the treating physician and the nursing team and sent to the PTCC. In case of ambulatory BT, clinical follow-up information was collected directly from the patients. All demographic, bacteriophage product, and clinical data were recorded in a REDCap (Research Electronic Data Capture) designed database. Details on settings, places where the data were collected and periods when collected and data collection are included in the manuscript.
Outcomes	Clinical improvement, eradication of the targeted bacterium, and the advent, severity and duration of adverse events or reactions were assessed by the treating physician.
